# Anti-Inflammatory Effects of Vitisinol A and Four Other Oligostilbenes from *Ampelopsis brevipedunculata* var. *Hancei*

**DOI:** 10.3390/molecules22071195

**Published:** 2017-07-17

**Authors:** Chi-I Chang, Wei-Chu Chien, Kai-Xin Huang, Jue-Liang Hsu

**Affiliations:** 1 Department of Biological Science and Technology, National Pingtung University of Science and Technology, Pingtung 900, Taiwan; changchii@mail.npust.edu.tw (C.-I.C.); omg740805@gmail.com (W.-C.C.) yhdscndsl@gmail.com (K.-X.H.); 2 Research Center for Tropic Agriculture, National Pingtung University of Science and Technology, Pingtung 900, Taiwan; 3 Research Center for Austronesian Medicine and Agriculture, National Pingtung University of Science and Technology, Pingtung 900, Taiwan

**Keywords:** *Ampelopsis brevipedunculata* var. *hancei*, anti-inflammatory effect, oligostilbenes, vitisinol A

## Abstract

In this study, the cytotoxicities and anti-inflammatory activities of five resveratrol derivatives—vitisinol A, (+)-ε-viniferin, (+)-vitisin A, (−)-vitisin B, and (+)-hopeaphenol—isolated from *Ampelopsis brevipedunculata* var. *hancei* were evaluated by 3-(4,5-dimethylthiazol-2-yl)-2,5-diphenyltetrazolium bromide (MTT) assay and lipopolysaccharide (LPS)-stimulated RAW264.7 cells, respectively. The result from MTT assay analysis indicated that vitisinol A has lower cytotoxicity than the other four well-known oligostilbenes. In the presence of vitisinol A (5 μM), the significant reduction of inflammation product (nitric oxide, NO) in LPS-induced RAW264.7 cells was measured using Griess reaction assay. In addition, the under-expressed inflammation factors cyclooxygenase-2 (COX-2) and inducible nitric oxide synthase (iNOS) in LPS-induced RAW264.7 cells monitored by Western blotting simultaneously suggested that vitisinol A has higher anti-inflammatory effect compared with other resveratrol derivatives. Finally, the anti-inflammatory effect of vitisinol A was further demonstrated on 12-*O*-tetradecanoylphorbol 13-acetate (TPA)-induced ear edema in mice. As a preliminary functional evaluation of natural product, the anti-inflammatory effect of vitisinol A is the first to be examined and reported by this study.

## 1. Introduction

Inflammation is the body’s protective response to injuries or infections caused by harmful stimuli such as pathogens, damaged cells, or irritants [1]. Acute inflammation is triggered by inflammatory mediators released by cells (e.g., macrophages and dendritic cells) already present in all tissues where injury or infection occurred. The inflammatory mediators include pro-inflammatory enzymes, cytokines, reactive oxygen species (ROS), nitric oxide (NO), and signaling proteins, and will lead to typical signs of inflammation (e.g., pain, swelling, edema, redness, and heat) in the infected or damaged tissues [2,3]. When tissues are under a long-term exposure to inflammatory mediators (known as chronic inflammation), the related organ systems may be affected and it may lead to chronic diseases such as cardiovascular diseases, Alzheimer’s disease, type 2 diabetes, Crohn’s disease and ulcerative colitis, asthma, or rheumatoid arthritis [4]. Moreover, some epidemiological investigations have suggested that chronic inflammation is also associated with 15–20% of all cancer related deaths and concluded that chronic inflammation can be regarded as a major risk factor for various types of cancer [2,5]. To relieve or avoid the effects caused by chronic inflammation, several types of synthetic drugs have been developed for blocking the inflammatory signaling pathway based on molecular targets such as lipoxygenases (LOXs), cyclooxygenase (COX), nuclear factor (NF)-κB, and tumor necrosis factor (TNF)-α in the inflammation cascades [6]. Due to the inspiration of the first anti-inflammatory natural product acetylsalicylic acid (aspirin) successfully introduced to treat rheumatic diseases in 1899, the natural products derived from traditional medicinal plants have attracted scientists’ attention and offer great promise to uncover bioactive natural products for the treatment or chemoprevention of inflammatory diseases [7]. The anti-inflammatory effects of various types of natural products, including alkaloids, fatty acids, steroids, triterpenoids, stilbenes, and flavonoids, have been extensively reported in several reviews [8,9,10,11]. Among these natural products, resveratrol—which was first isolated and characterized from the roots of white hellebore *Veratrum grandiflorum* in 1940—has been of great scientific interest in its anti-inflammatory activity over the past decades [12]. More recently, Wang et al. demonstrated the anti-inflammatory effects of resveratrol and some oligostilbenes, such as (+)-ε-viniferin, ampelopsin C, ampelopsin A, (−)-vitisin B, and (+)-vitisin A, isolated from *Vitis thunbergii* var. *taiwaniana* toward lipopolysaccharide-induced arthritis [13]. Nassra et al. further studied the anti-inflammatory effects of twenty-five stilbenoids and oligostilbenes isolated from *Milicia excelsa*, *Morus alba*, *Gnetum africanum*, and *Vitis vinifera*. Their data indicated that stilbene tetramers can reduce lipopolysaccharide-induced nitric oxide production more significantly than one trimer (E-miyabenol C), twelve dimers, and eight monomers [14]. Interestingly, the tetramers were only isolated from *Vitis vinifera* but not from the other three plants. However, the most effective tetramers hopeaphenol and isohopeaphenol were also accompanied with significant cytotoxicity at 5 and 10 μM, which dramatically limits their clinical applications. Nevertheless, the less toxic and less effective (−)-vitisin B still can effectively inhibit NO production from lipopolysaccharide (LPS)-induced BV-2 microglial cells with an IC_50_ value as low as 4.7 ± 0.5 μM, which shows better anti-inflammatory effect than resveratrol (IC_50_ = 13.1 ± 1.3 μM) [14].

**Figure 1:**
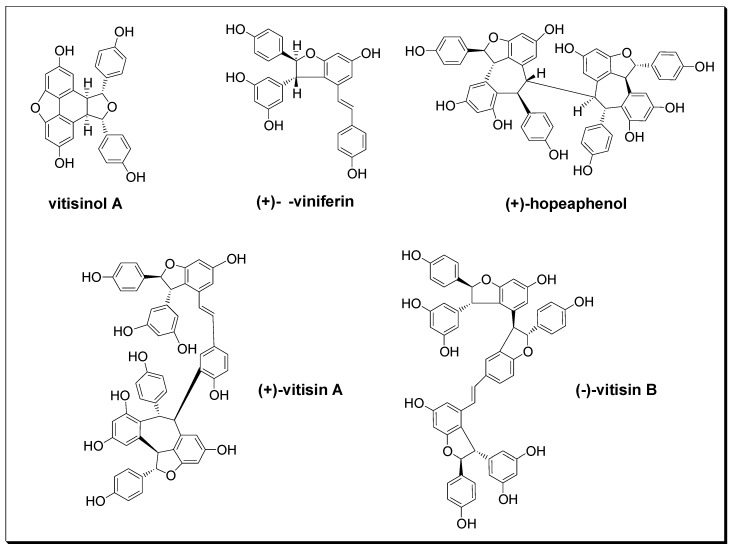
Chemical structures of five oligostilbenes.

*Ampelopsis brevipedunculata* (Maxim.) Traut. var. *hancei* (Planch.) Rehder (ABH) is a perennial climbing woody-stemmed plant widely distributed from the ground to the low altitude areas of Taiwan, and has long been used in traditional medicine for the treatment of rheumatoid arthritis, hepatitis, nephritis, and hypertension in Taiwan [15]. According to the previous study reported by Su et al. [16], ten resveratrol derivatives were isolated from this plant, and their angiotensin I converting enzyme (ACE) inhibitory activities were comprehensively screened. Their data suggested that ABH’s antihypertension effect is mainly contributed by (+)-vitisin A and (+)-hopeaphenol due to their remarkable ACE inhibitory activities [16]. However, the natural component which contributes ABH’s anti-inflammatory effect has not been well identified. Among dozens of oligostilbenes isolated from ABH, vitisinol A was first purified from *Vitis thunbergii* roots and characterized by Huang et al. in 2005 [17]. However, except for its ACE inhibitory activity studied by Su et al. [16], no other biological activities of vitisinol A have been examined and reported. In this study, five stilbene-type compounds (as shown in Figure 1) were isolated from dried ABH slices of whole plant according to the previous report [16]. Their structures were characterized using ^1^H and ^13^C nuclear magnetic resonance (NMR) spectroscopy, Fourier transform infrared (FT-IR) spectroscopy, mass spectrometry (MS), and optical rotation, as shown in the Supporting Information. The identities of these stilbene-type compounds (vitisinol A, (+)-ε-viniferin, (+)-hopeaphenol, (+)-vitisin A, and (−)-vitisin B) were further confirmed by comparing to those data reported in the literature [17,18,19,20]. Their abundances in different parts of ABH were also determined previously [16]. Among these oligostilbenes, vitisinol A and (+)-ε-viniferin are classified as resveratrol dimers; (+)-vitisin A, (−)-vitisin B, and (+)-hopeaphenol can be regarded as resveratrol tetramers. Notably, vitisinol A is a meso compound which has an internal plane of symmetry, while the others are chiral compounds. The health effects of (+)-ε-viniferin, (+)-hopeaphenol, (+)-vitisin A, and (−)-vitisin B have been widely reported [21,22,23,24,25], but the biological activity of vitisinol A has not been well studied since it was isolated from *Vitis thunbergii* roots and its structure was characterized by Huang et al. in 2005 [17]. The only known activity derived from vitisinol A is its moderate ACE inhibitory activity (IC_50_ ~ 8 μM) reported by Su et al. [16]. In their study, the abundances of vitisinol A in different parts of ABH were also determined using liquid chromatography-tandem mass spectrometry (LC-MS/MS) under a selective reaction monitoring (SRM) mode (see supplementary material). According to their result, the abundance of vitisinol A in the bark of ABH was measured as 3.63 ± 0.46 (μg/g dried weight)—twice higher than that in root, stem, or leaf [16]. In the present study, the cytotoxicity of vitisinol A was examined using MTT assay, and its anti-inflammatory activity was evaluated using NO production and the expression profiling of COX-2 and iNOS from LPS-stimulated RAW264.7 macrophages. Meanwhile, this vitisinol A compound’s health benefits towards inflammation were also compared with four other oligostilbenes which were also isolated from ABH: (+)-ε-viniferin, (+)-vitisin A, (−)-vitisin B, and (+)-hopeaphenol. Furthermore, the topical anti-inflammatory effect of vitisinol A was demonstrated in the model of 12-*O*-tetradecanoylphorbol 13-acetate (TPA)-induced ear inflammation in ICR (Institute for Cancer Research) mice.

## 2. Results & Discussions

### 2.1. Cytotoxicity Effects of Five Oligostilbenes against RAW 264.7 Cells

**Figure 2:**
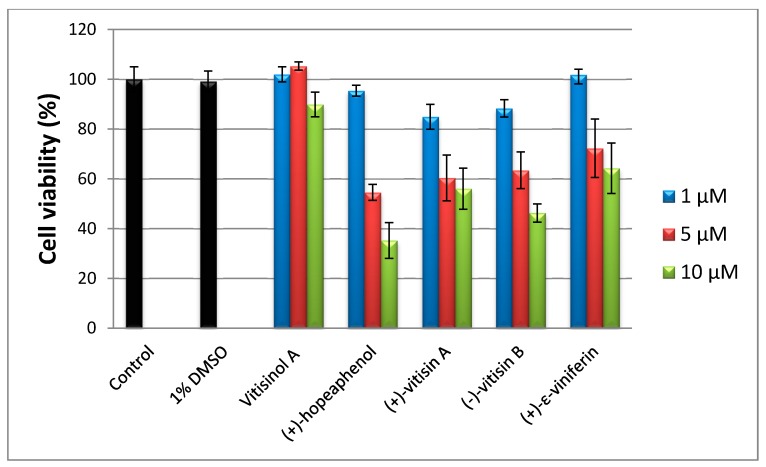
3-(4,5-dimethylthiazol-2-yl)-2,5-diphenyltetrazolium bromide (MTT) assay of RAW264.7 cells treated with five oligostilbenes at 1.5 and 10 μM.

To evaluate the cytotoxicities of five oligostilbenes towards RAW 264.7 cells, an 3-(4,5-dimethylthiazol-2-yl)-2,5-diphenyltetrazolium bromide (MTT) assay was performed. Since the natural products were dissolved in 0.1% DMSO, control was run in presence of 0.1% DMSO to eliminate the cytotoxicity effect caused by the solvent. In the medium of 10 μM of vitisinol A, the cell viability was around 90%; the other four oligostilbenes (at 10 μM) dramatically reduced cell viability to 60% or lower, which indicates that vitisinol A is less toxic than the other four oligostilbenes, as shown in Figure 2. According to the previous report [14], tetramers (+)-hopeaphenol and (+)-vitisin A showed significant cytotoxicity towards BV-2 microglial cells at 10 μM, while (+)-ε-viniferin and (−)-vitisin B were less toxic than (+)-hopeaphenol. In that study, (+)-hopeaphenol showed the highest inhibitory activity on NO production, but accompanied with the lowest cell viability in LPS-stimulated BV-2 microglial cells. In another cytotoxicity test study, Muhtadi et al. also found that hopeaphenol showed the highest cytotoxicity against murine leukemia P-388 cells when compared with the five known resveratrol oligomers diptoindonesin E, (+)-ε-viniferin, laevifonol, α-viniferin, and vaticanol B [26], which also highlighted (+)-hopeaphenol’s unignorable cytotoxicity. In this investigation, hopeaphenol at 10 μM significantly reduced the viability of RAW 264.7 cells to ~35%, which showed a similar trend as those findings reported by [14]. Notably, vitisinol A showed a negligible cytotoxicity even at 10 μM when compared with (+)-ε-viniferin and (−)-vitisin B, which were regarded as less toxic against BV-2 microglial cells [14]. The finding of relatively low cytotoxicity of vitisinol A against RAW 264.7 cells is the first to be discovered and reported by this study.

### 2.2. Anti-Inflammatory Effects of Five Oligostilbenes Against RAW 264.7 Cells

**Figure 3:**
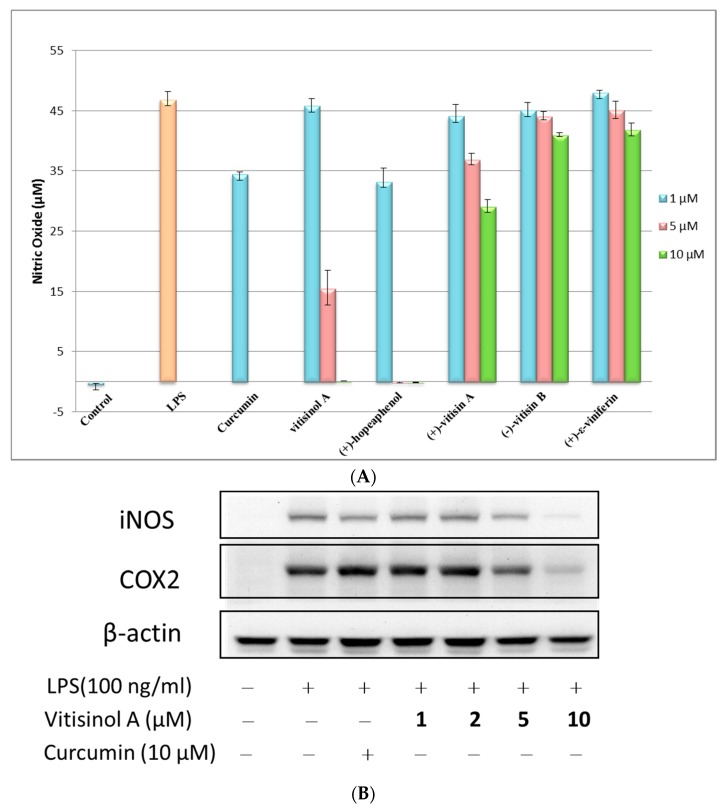
Anti-inflammatory effect of vitisinol A and its comparison with other four resveratrol oligomers. (**A**) Effect of five oligostilbenes on nitric oxide (NO) production at 1.5 and 10 μM in lipopolysaccharide (LPS)-activated RAW264.7 cells; (**B**) Effect of vitisinol A on inducible nitric oxide synthase (iNOS) and cyclooxygenase-2 (COX2) expression in LPS-induced RAW 264.7 macrophage cells. The experimental details are shown in Materials & Methods.

The anti-inflammatory effects of these five oligostilbenes were further evaluated using LPS-treated Raw 264.7 cells. The NO reduction experiment indicated that (+)-hopeaphenol and vitisinol A at 10 μM dramatically decreased the NO production of LPS-stimulated RAW 264.7 cells, while (+)-vitisin A showed moderate inhibitory effect and (+)-ε-viniferin and (−)-vitisin B did not show significant anti-inflammatory effects, as shown in Figure 3A. The trend of NO reduction effect for (+)-hopeaphenol, (+)-vitisin A, (+)-ε-viniferin, and (−)-vitisin B against RAW 264.7 cells was very similar to that towards BV-2 microglial cells [14]. According to the result of cytotoxicity test, hopeaphenol significantly reduced the viability of RAW 264.7 cells, even at 5 μM. Therefore, the significant NO reduction caused by (+)-hopeaphenol may be partly contributed by cell death due to its high cytotoxicity. Notably, the less-toxic vitisinol A also showed a significant NO reduction effect at 5 μM (~66%) and 10 μM (~100%), which implied that vitisinol A could be a more promising and effective anti-inflammatory agent than the other four resveratrol oligomers. To further examine the anti-inflammatory effect of vitisinol A, the expression of inflammation factors cyclooxygenase-2 (COX-2) and inducible nitric oxide synthase (iNOS) in LPS-induced RAW264.7 cells was further monitored by Western blotting. In Figure 3B, the expressions of COX-2 and iNOS simultaneously increase when LPS (100 ng/mL) was added in RAW264.7 cells. Meanwhile, their expressions were decreased dose-dependently in the presence of vitisinol A at 1, 2, 5 and 10 μM, respectively. The inflammation factors COX-2 and iNOS were almost diminished to a basal level in the presence of 10 μM of vitisinol A, which indicated its potent effect on suppressing these inflammatory mediators. According to previous study, the resveratrol tetramer (+)-vitisin A also can suppress LPS-induced NO production by inhibiting ERK, p38, and NF-κB activation in RAW 264.7 cells [27]. In our study, the resveratrol dimer vitisinol A showed lower cytotoxicity and higher anti-inflammatory effect compared to that of (+)-vitisin A.

### 2.3. Anti-Inflammation Effect of Vitisinol A on TPA-Induced Ear Edema of Mice

**Figure 4:**
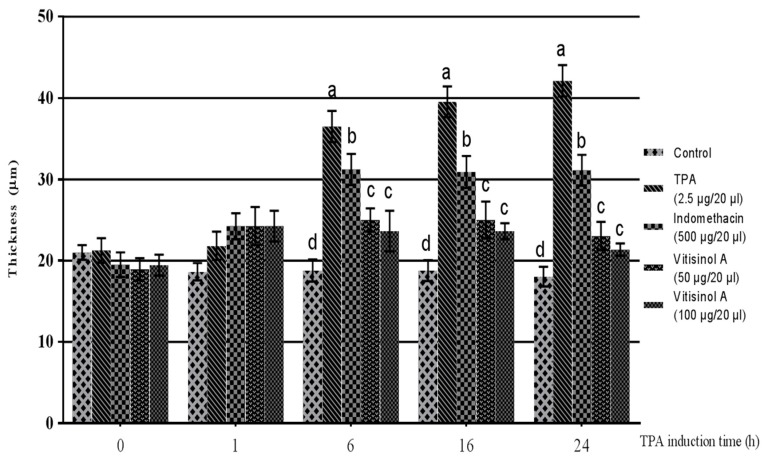
The anti-inflammatory effect of vitisinol A on 12-*O*-tetradecanoylphorbol 13-acetate (TPA)-induced ear edema of mice. The mice were divided into five groups: Control (without TPA and vitisinol A treatment), TPA (TPA-induced ear), Indomethacin (500 μg of indomethacin on TPA-induced ear), and Vitisinol A (50 or 100 μg of vitisinol A on TPA-induced ear). The experimental details are shown in Materials & Methods. Different letters indicate the significant difference between groups (*p* < 0.05, Duncan test).

The in vitro data indicated that the less-cytotoxic vitisinol A can dramatically reduce inflammatory mediators such as NO, COX-2, and iNOS. The anti-inflammatory effect of vitisinol A was further confirmed using an in vivo model of TPA-induced ear edema of mice. Before TPA treatment, the ear thicknesses of five groups did not show significant difference. After 1 h of TPA treatment, the TPA-pretreated right ears for four groups were applied with acetone (20 μL/ear, denoted as TPA), positive control indomethacin (500 μg dissolved in 20 μL of acetone/ear), vitisinol A (50 μg dissolved in 20 μL of acetone/ear), and vitisinol A (100 μg dissolved in 20 μL of acetone/ear), respectively. The TPA-treated ears became swollen and the ear thickness dramatically increased after 6 h, and the ear edema lasted even for 24 h. Notably, the ear thickness of the vitisinol A-treated group (at either 50 μg/20 μL or 100 μg/20 μL) was significantly decreased compared with the only TPA-treated group, which indicated that the ear edema was relieved in the presence of vitisinol A, as shown in Figure 4. Moreover, the anti-inflammatory effect of vitisinol A at a dose of 50 μg was even better than indomethacin (at a dose of 500 μg), a commercial nonsteroidal anti-inflammatory drug (NSAID) [28]. The anti-inflammatory effect of resveratrol oligomers towards TPA-induced ear edema of mice has not been well studied. According to a previous report, resveratrol monomer (0.62 mg) was demonstrated to produce an effect similar to indomethacin (0.5 mg) in the model of TPA-induced ear edema [29]. Therefore, we concluded from our analysis and findings that the anti-inflammatory effect of vitisinol A was much higher than resveratrol and other resveratrol oligomers.

## 3. Materials and Methods

### 3.1. Raw Materials and Chemical Reagents

3-(4,5-dimethylthiazol-2-yl)-2,5-diphenyltetrazolium bromide (MTT), indomethacin, 12-*O*-tetradecanoylphorbol 13-acetate (TPA), curcumin, *N*,*N*,*N*,*N*-tetramethyl-ethylenediamine (TEMED), phosphate-buffered saline (PBS), dimethyl sulfoxide (DMSO), ammonium persulfate (APS), protease inhibitor cocktail, sodium dodecyl sulfate (SDS), NP-40, and DMEM (Dulbecco’s Modified Eagle Medium) were purchased from Sigma-Aldrich (St. Louis, MO, USA). Coomassie blue, Tris base, and Tris hydrochloride (Tris-HCl) were provided by J.T Baker (Phillipsburg, NJ, USA). Fetal Bovine Serum (FBS) and Dulbecco’s modified Eagle’s medium (DMEM) were obtained from Gibco (Grand Island, NY, USA). COX-2 and β-actin-specific antibodies were from Cell Signaling Technology (Boston, MA, USA). Antibody against iNOS was from BD Biosciences (Franklin Lakes, CA, USA). All secondary antibodies were purchased from GE Healthcare Life Sciences (Piscataway, NJ, USA).

### 3.2. Cell Culture and Sample Treatment

Macrophage RAW 264.7 cells, provided by Bioresource Collection and Research Center (BCRC #60001) in Taiwan, were cultured in DMEM supplemented with fetal bovine serum (10%), and incubated at 37 °C in a humidified incubator supplied with 5% CO_2_. Cells were incubated with the tested samples (vitisinol A and other oligostilbenes) at indicated concentrations or curcumin (as positive control) for 1 h, then induced with LPS (100 ng/mL) for 12 h.

### 3.3. Cytotoxicity Assay

Cytotoxicities of oligostilbenes against RAW264.7 cells were evaluated using MTT assay. RAW264.7 cells (2 × 10^5^ cell/well) were seeded in a 96-well plate and treated with the PBS, DMSO, oligostilbenes in 1% DMSO diluted to the indicated concentrations in serum-free medium in the presence and absence of LPS (100 ng/mL) for 12 h. Here, DMSO (0.5%) was used to monitor its influence on the assays. The cells were then washed with PBS twice and immersed in 5 mg/mL MTT solution at 37 °C in the dark for 1 h. After that, DMSO (100 μL) was added to each well and incubated for 10 min, then the resulting supernatant was transferred to another 96-well plate, and the absorbance at 570 nm in each well was determined using a microplate reader (Molecular Devices, Sunnyvale, CA, USA).

### 3.4. Nitric Oxide Inhibitory Assay

RAW 264.7 cells were plated at 2 × 10^5^ cells/well in 24-well plates and then treated with LPS (100 ng/mL) in the absence and presence of oligostilbenes at indicated concentrations for 12 h. To study whether or not the LPS-induced nitric oxide was inhibited by these natural products, nitrite levels in culture media in the absence and presence of oligostilbenes were determined using the Griess reaction assay. Briefly, 100 μL of cell culture medium was mixed with 100 μL of Griess reagent (containing 0.1% naphthylethylenediamine-dihydrochloride and 1% sulfanilamide in 5% phosphoric acid), and the mixture stayed at r.t. for 10 min. Finally, the absorbance at 540 nm in each well was determined using a microplate reader (Versa-Max; Molecular Devices, CA, USA). In this assay, fresh medium without natural product was used as blank. The nitrite concentration in each sample was calculated according to the standard curve established using various concentrations of sodium nitrite.

### 3.5. Western Blot Analysis

RAW 264.7 cells seeded at a density of 4 × 10^6^/well in a 24-well plate were incubated with or without natural products (at indicated concentrations) and LPS (final concentration 100 ng/mL) for 12 h for the detection of the expressions of iNOS and COX-2. Here, curcumin (10 μM) was used as positive control. After treatment, cold PBS (pH 7.4) was added to wash the cells two times and the cells were immersed in lysis buffer (Promega, Madison, WI, USA). The suspension was centrifuged at 14,000 *g* at 4 °C for 15 min, then the supernatant was collected and the total protein concentration of the lysate was determined using the BCA Protein Assay kit (Pierce, IL, USA). Equal amounts of proteins were subjected to SDS-PAGE and Western blotting according to previous report with slight modifications [30]. Briefly, control and treated samples were separated on SDS-PAGE gels, and the resulting protein bands were transferred to PVDF membranes (Millipore) for 2 h at a fixed current. The membranes were then incubated with rabbit antibodies of COX-2 and β-actin and mouse antibody of iNOS at 4 °C for overnight. The membranes were washed using PBST (10 mM NaH_2_PO_4_, 130 mM NaCl, 0.05% Tween 20) three times, then the secondary antibodies (goat anti-rabbit) conjugated with horseradish peroxidase in blocking solution (1:5000) were added to recognize and probe the primary antibodies. Excess secondary antibodies and blocking solution were removed and the membranes were washed with PBST buffer. The immunoreactive bands were detected using UVP Biospectrum imaging system (Upland, CA, USA) and the relative band intensities were quantified using the supplied software.

### 3.6. Animal Tests

The protocols for in vivo experiments were approved by the Institutional Animal Care and Use Committee (IACUC) in National Pingtung University of Science and Technology (NPUST) in accordance with international guidelines. ICR mice (male, 30–35 g) used in this study were purchased from BioLASCO (Taipei, Taiwan). ICR mice were fed with regular laboratory diet and water ad libitum and housed under a 12-h light–dark cycle. TPA-induced ear edema of mice was performed based on the previous report with slight modifications [31]. TPA (2.5 μg dissolved in 20 μL of acetone) was topically applied to the inner and outer surfaces of the right ears of mice. After 1 h, the mice were divided into four groups (six mice for each group), acetone was applied to the left ears of all mice in the four groups (20 μL/ear), as normal control (denoted as Control). Meanwhile, the TPA-pretreated right ears for four groups were applied acetone (20 μL/ear, denoted as TPA), positive control indomethacin (500 μg dissolved in 20 μL of acetone/ear, denoted as Indomethacin), vitisinol A (50 μg dissolved in 20 μL of acetone/ear, denoted as vitisinol A 50 μg/20 μL), and vitisinol A (100 μg dissolved in 20 μL of acetone/ear, denoted as vitisinol A 100 μg/20 μL), respectively. The thickness of ears was measured before and at 1 h, 6 h, 16 h, and 24 h after TPA treatment using a dial thickness gauge (Peacock, Tokyo, Japan).

### 3.7. Statistical Analysis

Data were pooled from three to six independent experiments and expressed as the mean ± SE. The data were evaluated via one-way ANOVA using SPSS program (version 19. SPSS Inc., Endicott, NY, USA). Duncan’s multiple range tests were performed using GraphPad Software 5.0 (San Diego, CA, USA) to analyze the differences between the TPA control and the experimental groups at the same time. The result was regarded as significantly different when the resulting *p*-value was smaller than 0.05.

## 4. Conclusions

We demonstrated that the cytotoxicity of vitisinol A derived from *Ampelopsis brevipedunculata* var. *hancei* (Planch.) Rehder was relatively lower than the other four well-known resveratrol oligomers ε-viniferin, hopeaphenol, vitisin A, and vitisin B. In addition, the NO reduction and suppressed expression of COX-2 and iNOS in LPS-induced RAW264.7 cells simultaneously indicated that vitisinol A is a potent anti-inflammatory agent in vitro. Moreover, the in vivo anti-inflammatory effect of vitisinol A was further examined and confirmed using the model of TPA-induced ear edema of mice. Although the detailed mechanism of vitisinol A involved in either in vitro or in vivo anti-inflammation remains to be explored, the preliminary findings from our study informatively suggest that less-cytotoxic vitisinol A may be a potential therapeutic agent for the relief of inflammatory symptoms.

## Supplementary Materials

Spectral data of Vitisinol A and four other oligostilbenes.
