# Supplementary material for: Anti-Inflammatory Effects of Vitisinol A and Four Other Oligostilbenes from Ampelopsis brevipedunculata var. Hancei
Source: Molecules. 2017 Jul 17;22(7):1195. doi: 10.3390/molecules22071195 (PMC6152071; doi:10.3390/molecules22071195)
Supplement: Supplementary file 1 [file molecules-22-01195-s001.pdf]

molecules

1. Spectral data

Vitisinol A (1): brown powder; UV λ_{max} (MeOH): 278 (3.40), 232 (3.65) nm; FAB-MS *m/z*: 452 $[M]^+$; ^{1}H-NMR (400 MHz, acetone-*d*$_6$): δ 7.59 (4H, d, *J* = 8.8 Hz, H-2a, 2b, 6a, 6b), 6.97 (4H, d, *J* = 8.8 Hz, H-3a, 3b, 5a, 5b), 6.19 (2H, d, *J* = 2.0 Hz, H-14a,14b), 6.17 (2H, d, *J* = 2.0 Hz, H-12a, 12b), 5.49 (2H, d, *J* = 10.8 Hz, H-7a, 7b), 4.49 (2H, d, *J* = 10.8 Hz, H-8a, 8b); ^{13}C-NMR (100 MHz, acetone-*d*$_6$): δ 48.5 (d, C-8a, 8b), 93.0 (d, C-7a, 7b), 96.9 (d, C-12a, 12b), 104.3 (d, C-14a, 14b), 116.6 (d, C-3a, 3b, 5a, 5b), 122.3 (s, C-10a, 10b), 130.3 (d, C-2a, 2b, 6a, 6b), 131.3 (s, C-1a, 1b), 137.0 (s, C-9a, 9b), 159.1 (s, C-4a, 4b), 159.7 (s, C-11a, 11b), 160.1 (s, C-13a, 13b).

(+)-ε-viniferin Brown solid; $[\alpha]_D^{25}$ = +36.2 (*c* = 0.51, MeOH); UV λ_{max} (MeOH): 285 (3.98), 319 (4.10) nm; FAB-MS *m/z*: 454 $[M]^+$; ^{1}H-NMR (400 MHz, acetone-*d*$_6$): δ 4.47 (1H, d, *J* = 5.6 Hz, H-8a), 5.42 (1H, d, *J* = 5.6 Hz, H-7a), 6.24 (each 1H, br s, H-10a, 12a, 14a), 6.33 (1H, d, *J* = 1.6 Hz, H-12b), 6.71 (1H, d, *J* = 16.4 Hz, H-8b), 6.73 (each 1H, d, *J* = 8.4 Hz, H-3b, 5b), 6.83 (2H, d, *J* = 8.4 Hz, H-3a, 5a), 6.91 (1H, d, *J* = 16.4 Hz, H-7b), 7.17 (each 1H, d, *J* = 8.4 Hz, H-2b, 6b), 7.20 (each 1H, d, *J* = 8.4 Hz, H-2a, 6a); ^{13}C-NMR (100 MHz, acetone-*d*$_6$): δ 57.2 (d, C-8a), 94.0 (d, C-7a), 96.9 (d, C-12b), 102.2 (d, C-12a), 104.3 (d, C-14b), 107.1 (d, C-10a, -14a), 116.3 (d, C-3a, -5a), 116.4 (d, C-3b, -5b), 119.9 (s, C-10b), 123.5 (d, C-8b), 128.0 (d, C-2a, -6a), 128.8 (d, C-2b, -6b), 130.0 (s, C-1b), 130.2 (d, C-7b), 133.9 (s, C-1a), 136.5 (s, C-9b), 147.5 (s, C-9a), 158.3 (s, C-4a), 159.7 (s,C-13b), 160.0 (s, C-11a, -13a), 162.5 (s, C-11b).

(+)-hopeaphenol: brown-yellow powder; $[\alpha]_D^{25}$ +390.0° (c 0.35, MeOH); ESI-MS *m/z* : 907 $[M+H]^+$; ^{1}H-NMR (400 MHz, Acetone-*d*$_6$): δ 7.12 (2H, d, *J* = 8.4 Hz, H-2a, 6a), 6.89 (2H, d, *J* = 8.4 Hz, H-2b, 6b), 6.77 (2H, d, *J* = 8.8 Hz, H-3a, 5a), 6.54 (2H, d, *J* = 8.8 Hz, H-3b, 5b), 6.28 (2H, d, *J* = 2.4 Hz, H-14a), 5.80 (1H, brs, H-7b), 5.73 (1H, d, *J* = 12.0 Hz, H-7a), 5.71 (1H, d, *J* = 2.0 Hz, H-12b), 5.15 (1H, d, *J* = 2.0 Hz, H-14b), 4.22 (1H, d, *J* = 12.0 Hz, H-8a), 3.93 (1H, d, *J* = 4.8 Hz, H-7b).

(+)-vitisin A: brown solid; $[\alpha]_D^{25}$ = +210.5° (c 0.4, MeOH); ESI-MS *m/z*: 907$[M+H]^+$; ^{1}H-NMR (400 MHz, Acetone-*d*$_6$): δ 7.19 (2H, d, *J* = 8.5 Hz, H-2a, 6a), 7.14 (2H, d, *J* = 8.5 Hz, H-2c, H-6c), 7.02 (2H, d, *J* = 8.3 Hz, H-2d, H-6d), 6.87 (1H, dd, *J* = 8.4, 2.2 Hz, H-6b), 6.81 (2H, d, *J* = 8.5 Hz, H-3a, 5a), 6.77 (2H, d, *J* = 8.5 Hz, H-3c, H-5c), 6.68 (1H, d, *J* = 8.4 Hz, H-5b), 6.65 (2H, d, *J* = 8.3 Hz, H-3d, H-5d), 6.50 (1H, d, *J* = 2.2 Hz, H-14b), 6.38 (2H, brs, H-7b, H-8b), 6.25 (1H, d, *J* = 2.2 Hz, H-12b), 6.23 (1H, d, *J* = 2.2 Hz, H-14c), 6.21 (1H, d, *J* = 2.2 Hz, H-12a), 6.16 (2H, d, *J* = 2.2 Hz, 10a, 14a), 6.08 (1H, d, *J* = 1.8 Hz, H-12d),

6.07 (1H, d, *J* = 2.2 Hz, H-2b), 6.04 (1H, d, *J* = 1.8 Hz, H-14d), 6.02 (1H, d, *J* = 2.2 Hz, H-12c), 5.87 (1H, d, *J* = 11.6 Hz, H-7c), 5.47 (1H, d, *J* = 3.3 Hz, H-8d), 5.38 (1H, d, *J* = 3.3 Hz, H-7d), 5.35 (1H, d, *J* = 5.3 Hz, H-7a), 4.40 (1H, d, *J* = 5.3 Hz, H-8a), 4.23 (1H, d, *J* = 11.6 Hz, H-8c).

(−)-vitisin B: brown solid; $[\alpha]_D^{25} = -112.4°$ (c 0.5, MeOH); ESI-MS *m*/*z*: 907 $[M+H]^+$; ^{1}H-NMR (400 MHz, Acetone-d_6): 7.26 (2H, d, *J* = 8.6 Hz, H-2a, H-6a), 7.20 (2H, d, *J* = 8.5 Hz, H-2d, H-6d), 7.14 (1H, d, *J* = 8.3 Hz, H-6b), 6.92 (2H, d, *J* = 8.6 Hz, H-3a, H-5a), 6.83 (2H, d, *J* = 8.6 Hz, H-3d, H-5d), 6.76 (1H, d, *J* = 16.8 Hz, H-8b), 6.70–6.62 (5H, m, H-5b, H-2b, H-14b, H-2c, H-6c), 6.61 (1H, d, *J* = 16.8 Hz, H-7b), 6.59 (2H, d, *J* = 8.6 Hz, H-3c, H-5c), 6.33 (2H, brs, H-12c), 6.24–6.19 (5H, m, H-12b, H-10d, H-14d, H-12d, H-14c, H-12a), 6.13 (2H, d, *J* = 2.2 Hz, H-10a, H-14a), 5.55 (1H, d, *J* = 4.7 Hz, H-7c), 5.41 (2H, m, H-7d, H-7a), 4.53 (1H, d, *J* = 4.5 Hz, H-8a), 4.45 (1H, d, *J* = 5.4 Hz, H-8d), 4.33 (1H, d, *J* = 4.7 Hz, H-8c).

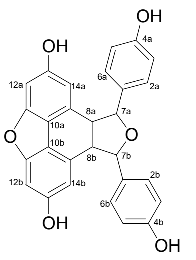

vitinsinol A

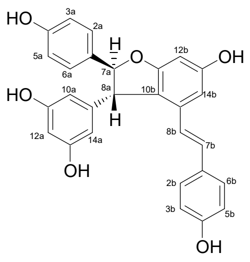

(+)-ε-viniferin

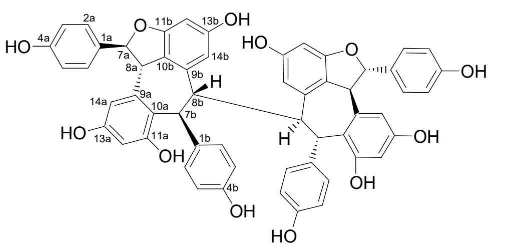

(+)-hopeaphenol

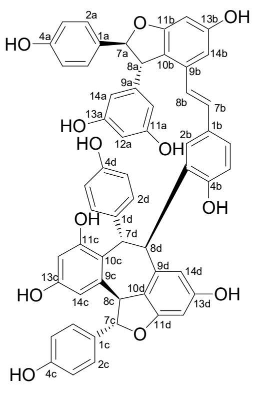

(+)-vitisin A

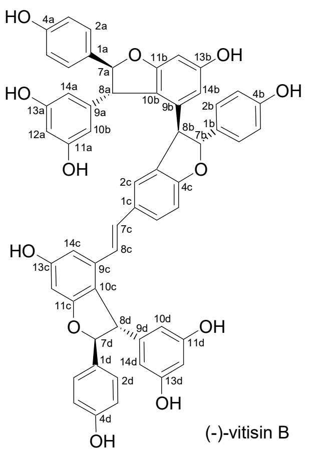

(-)-vitisin B
